# Cytokine Effects on Cell Viability and Death of Prostate Carcinoma Cells

**DOI:** 10.1155/2014/536049

**Published:** 2014-05-29

**Authors:** Georgios Chondrogiannis, Michalis Kastamoulas, Panagiotis Kanavaros, Georgios Vartholomatos, Maria Bai, Dimitrios Baltogiannis, Nikolaos Sofikitis, Dimitrios Arvanitis, Vasiliki Galani

**Affiliations:** ^1^Department of Anatomy-Histology-Embryology, Medical School, University of Ioannina, 45110 Ioannina, Greece; ^2^Laboratory of Hematology, University Hospital of Ioannina, 45500 Ioannina, Greece; ^3^Department of Pathology, Medical School, University of Ioannina, 45110 Ioannina, Greece; ^4^Department of Urology, Medical School, University of Ioannina, 45110 Ioannina, Greece; ^5^Department of Anatomy, Medical School, University of Thessaly, 44110 Larisa, Greece

## Abstract

We analyzed the effects of IL-13, IFN-**γ**, and IL-1**β** on cell viability and death of LNCaP and PC-3 cells and major signaling pathways involved in these effects. Significant increase of LNCaP cell death (apoptotic and necrotic) and increased levels of active caspase 3 were observed in cells treated with inhibitors of ERK 1/2 (UO126) and p38 (SB203580) prior to IL-1**β** treatment in comparison to cells treated with UO126, SB203580, or IL-1**β** alone. Significant increase of LNCaP but not PC-3 cell death was detected after treatment with LY-294002 (inhibitor of phosphatidylinositol 3-kinase). No significant increase of LNCaP and PC-3 cell death was observed after treatment with SP600125 (inhibitor of JNK), SB203580 (inhibitor of p38), UO126 (inhibitor of ERK 1/2), or BAY 11-7082 (inhibitor of NF-**κ**B). Reduced c-FLIP_L_ expression was observed in LNCaP cells treated with LY-294002. The significant potentiation of LNCaP cell death by inhibition of ERK 1/2, p38, and PI3-K pathways may provide a rationale for therapeutic approach in androgen-dependent prostate cancer.

## 1. Introduction

Prostate cancer is the most frequently diagnosed malignant tumor among men and the second leading cause of male cancer-related mortality [1]. The growth and death of prostate cancer cells may be regulated by pathways involving Death Receptors such as TNF-related apoptosis inducing ligand- (TRAIL)-receptor and Fas/CD95 as well as by growth factors and cytokines which are secreted by tumor cells and/or immune cells of the tumor microenvironment [2–7].

Prostate carcinoma cell lines such as LNCaP, PC-3, DU-145, and M12 have been used as model systems to examine the effects of various cytokines on growth and death of prostate cancer cells. Indeed, TNF-*α* but not TRAIL treatment increased apoptosis in LNCaP cells whereas TRAIL but not TNF-*α* treatment increased apoptosis in PC-3 cells [8–13]. Moreover, IFN-*γ* but not IFN-*α* and IFN-*β* reduced cell viability and increased apoptosis in M12 cells, as determined by MTT and ELISA assays, respectively [14]. Furthermore, IL-13 and IL-1*β* reduced LNCaP cell viability and IL-13 reduced PC-3 cell viability, as determined by MTT or TTC assays [15–17]. However, the effects of IL-13, IFN-*γ*, and IL-1*β* on LNCaP and PC-3 cell death, to the best of our knowledge, have not been systematically analyzed by flow cytometry.

Major signaling pathways regulating cell growth and death such as nuclear factor-*κ*B (NF-*κ*B) [18], phoshatidylinositole-3 kinase (PI-3 K)/Akt [19, 20], and mitogen-activated protein kinases (MAPK) [21] are activated in prostatic carcinomas [7, 10, 21–30]. There is evidence that the aforementioned pathways may be regulated by cytokines and growth factors in prostate cancer. Indeed, TNF-*α* and IL-1*β* activate NF-*κ*B in LNCaP cells but TNF-*α* does not affect the constitutively activated NF-*κ*B in PC-3 cells [9, 11, 17, 31, 32]. Moreover, IL-1*β* activates the MAPK p38, extracellular signal regulated kinase (ERK 1/2) and c-jun NH2-terminal kinase (JNK) in DU-145 cells, treatment of PC-3 cells while TNF-*α* does not induce significant alterations in ERK 1/2, p38, and JNK phosphorylation and p38 activation by TNF-*α* protects LNCaP cells from apoptosis [10, 33]. However, the involvement of MAPK, PI3-K/Akt, and NF-*κ*B signaling pathways in IL-13, IFN-*γ*, and IL-1*β* effects on LNCaP and PC-3 cell death, to the best of our knowledge, has not been systematically analyzed.

Therefore, we analyzed (a) the effects of IL-13, IFN-*γ*, and IL-1*β* on cell viability, cycle and death of LNCaP, and PC-3 cells and (b) the involvement of MAPK, PI3-K/Akt, and NF-*κ*B signaling pathways in these cytokine-induced cellular effects. TNF-*α* with known procell death effects on LNCaP but not PC-3 cells [10, 11] was used as control.

## 2. Materials and Methods

### 2.1. Cell Culture

LNCaP (CRL-1740) and PC-3 (CRL-1435) human prostate carcinoma cells were obtained from ATCC and were used within six months of receipt. Cells were cultured in a 37°C, 5% CO_2_ humidified incubator in RPMI 1640 medium (Life Technologies Inc. A10491) or Ham's F12 K medium (Gibco 21127-022), respectively, supplemented with 10% heat-inactivated fetal bovine serum (FBS; Gibco 10270-106) and 1% antibiotic-antimycotic (Gibco 15240-062). Cells were passaged at 70–80% confluenceusing 1x Trypsin-EDTA (Gibco 15400-054).

### 2.2. Treatment with IL-13, IFN-*γ*, IL-1*β*, TNF-*α*, and Inhibitors of Various Signaling Pathways

LNCaP and PC-3 cells were plated at a concentration of 5 × 10^5^cells per well in 6-well tissue culture plates for 32 and 24 hours, respectively, in complete medium. Cells were then serum-starved for 24 h and growth arrested LNCaP and PC-3 cells were either untreated (control) or treated with IL-13, IFN-*γ*, IL-1*β*, or TNF-*α* (all from Sigma), with or without pretreatment with inhibitors of various signaling pathways. Inhibitors of NF-*κ*B (BAY-117082) and ERK 1/2 MAPK (UO126) pathways were purchased from Calbiochem, inhibitors of PI3-K (LY-294002) and p38 MAPK (SB203580) pathways were purchased from Sigma, and inhibitor of JNK (SP600125) pathway was purchased from Alexis Biochemicals. The incubation times and the concentrations of the cytokines and the chemical inhibitors used in the present study were chosen on the basis of previous studies [8, 10, 11, 15, 17, 22, 27, 31, 33, 34].

### 2.3. Methylthiazolyldiphenyl-Tetrazolium Bromide (MTT) Assay

LNCaP and PC-3 cells were seeded in 96-well plates at a concentration of 5 × 10^3^ cells per well in complete culture medium. After 32 h for LNCaP and 24 h for PC-3 cells the medium was changed to serum-free RPMI 1640 and Ham's F12 K medium, respectively, for 24 h before cells were treated with the indicated reagent. Then, the MTT** (**Methylthiazolyldiphenyl-tetrazolium bromide, Sigma Life Sciences) assay was performed as we previously described [35].

### 2.4. Flow Cytometry

LNCaP and PC-3 cells were plated in 6-well tissue culture plates at a concentration of 5 × 10^5^cells per well in complete growth medium for 32 h for LNCaP and 24 h for PC-3 and then sustained growth arrest in growth medium without FBS for 24 h. Cells were stimulated with TNF-*α*, IL-1*β*, IL-13, and IFN-*γ* with or without chemical inhibitors of various signaling pathways. The experimental approach was performed as we previously described [35]. Healthy cells generate a typical cell cycle histogram and the sub-G1 fraction represents the percentage of cell death [36].

Flow cytometric quantification of apoptotic and viable cells with annexin V-FITCH/Propidium Iodide staining was also performed. LNCaP cells were cultured, treated, and harvested as described above and resuspended in Calcium Buffer. Cells were then stained with 5 *μ*L annexin V-fluorescein isothiocyanate (FITC) (annexin V-FITC, 556420, BD Pharmingen) and 5 *μ*L of PI (propidium iodide solution, P4864, Sigma). Samples were then incubated for 15 min at room temperature in the dark and 1 mL of Calcium Buffer was added. The cytometric analysis was performed in a Partec ML flow cytometer (CyFlow ML, Partec, Munster, Germany) and results were analyzed by Partec FloMax software. Experiments were performed in triplicate and the results were expressed as mean values with standard deviations.

### 2.5. Cell Extracts

Following treatment as described above, adherent cells were trypsinised and pelleted at 100 g for 5 min at 4°C. Supernatants were discarded and the cell pellets were resuspended in 300 *μ*L of RIPA buffer (Tris-Hcl pH 7.4, 50 mM, NaCl, 150 mM, EDTA 20 mM, sodium deoxycholate 25 mM, SDS 35 mM, Triton X-100 1%), containing 1 mM PMSF, 4 *μ*g/mL leupeptin, 4 *μ*g/mL aprotinin, and 4 *μ*g/mL pepstatin. Cell lysates were sonicated 5x for 10 seconds and then vortexed. Samples were then centrifuged at 16,000 ×g for 25 min at 4°C. The resulting supernatant or cell extract was analyzed for protein concentration by the Bradford method (Bio-Rad, Hercules, CA) and stored at −80°C until further use.

### 2.6. Western Blot

Equal amounts of total cell lysates (20–60 *μ*g) were mixed with Laemmli buffer, denatured by boiling, and separated by sodium dodecyl sulfate-polyacrylamide gel electrophoresis (SDS-PAGE) as we previously described [37]. Proteins were then transferred to nitrocellulose membranes (Hybond-C Extra membrane, Amersham Biosciences). All membranes were blocked for 2 h in Tris-buffered saline with 0.1% Tween 20 (TBS-T) with 5% nonfat milk at room temperature. Membranes were then incubated in their respective primary antibody.

The following primary antibodies were used: monoclonal mouse anti-Bcl-X_L_ 1 : 100 (sc-8392), monoclonal mouse anti-Bad 1 : 100 (sc-8044), polyclonal rabbit anti-Bid 1 : 100 (sc-11423), polyclonal rabbit anti-Bax 1 : 200 (sc-493), monoclonal mouse anti-FLIP_L/S_ 1 : 200 (sc-5276), monoclonal mouse anti-I*κ*B*α* 1 : 100 (sc-1643), monoclonal mouse anti-p-JNK 1 : 200 (sc-6254), polyclonal goat anti-c-IAP1 1 : 200 (sc-1867), polyclonal rabbit anti-c-IAP2 1 : 200 (sc-7944), monoclonal mouse anti-caspase 3 1 : 100 (sc-7272) (all from Santa Cruz Biotechnology, Inc.), monoclonal mouse anti-p-Akt 1 : 200 (4051S), monoclonal mouse anti-p-p44/42 MAPK 1 : 200 (ERK 1/2; 9106S), monoclonal mouse anti-p-p38 1 : 200 (9216S) (all from Cell Signaling), monoclonal mouse anti-Fas 1 : 500 (Millipore, #05-201), monoclonal mouse anti-Bcl-2 1 : 20 (Cell Marque, clone: 124), monoclonal rabbit anti-cyclin-D1 1 : 10 (Cell Marque, clone: SP4), monoclonal mouse anti-*β*-actin 1 : 5000 (Sigma, A5441) and monoclonal mouse anti-*α*-tubulin 1 : 5000 (Sigma, T5168). The primary antibodies were diluted in TBS-T and 5% nonfat milk and incubated overnight at 4°C. For mouse monoclonal antibodies, membranes were incubated with the secondary antibody IgG HRP-conjugated goat anti-mouse 1 : 2000 (sc-2004, Santa Cruz Biotechnology, Inc.), for goat polyclonal antibodies, with the secondary antibody IgG HRP-conjugated rabbit anti-goat 1 : 2000 (sc-2768, Santa Cruz Biotechnology Inc.), and, for rabbit polyclonal and monoclonal antibodies, with the secondary antibody IgG HRP-conjugated goat anti-rabbit 1 : 2000 (sc-2004, Santa Cruz Biotechnology, Inc.), all in TBS-T with 5% nonfat milk, for 1 h at room temperature. Proteins were visualized on X-ray films with the ECL Detection System (GE Healthcare Life Sciences) according to manufacturer's specifications.

### 2.7. Statistical Analysis

Student's *t*-test and Mann-Whitney *U* test were used for statistical analysis. The results were considered as statistically significant when *P* < 0.05. The programs IBM SPSS Statistics Release 20 and GraphPad Prism Release 5 were used for statistical analysis and graph plotting.

## 3. Results

### 3.1. MTT Assay

**Figure 1:**
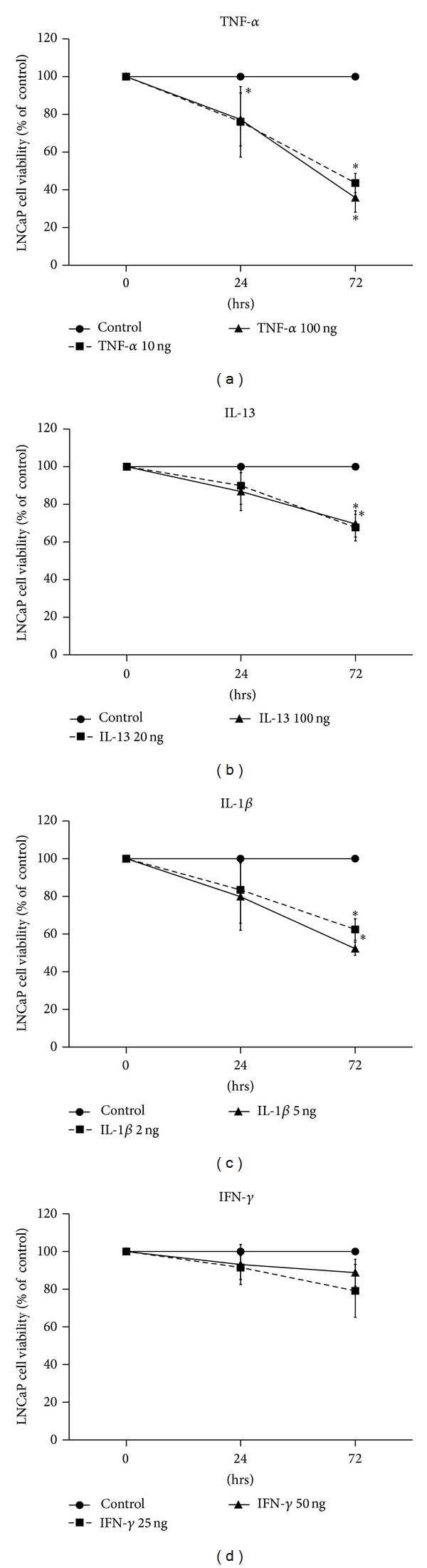
Cell viability assay of LNCaP cells using MTT. Time- and dose-dependent effects of (a) TNF-*α* (10 and 100 ng/mL), (b) IL-13 (20 and 100 ng/mL), (c) IL-1*β* (2 and 5 ng/mL), and (d) IFN-*γ* (25 and 50 ng/mL) on LNCaP cell viability, after treatment for 24 h or 72 h (*P < 0.05).

MTT assay was performed to analyze the LNCaP and PC-3 cell viability after treatment with IL-13, IFN-*γ*, IL-1*β*, or TNF-*α* (in 24 and 72 h). Treatment with TNF-*α* (10 and 100 ng/mL), IL-13 (20 and 100 ng/mL), IFN-*γ* (25 and 50 ng/mL), or IL-1*β* (2 and 5 ng/mL) for 24 and 72 h resulted in decreased cell viability of LNCaP cells in comparison to control cells (ctrl) (Figure 1).

A notable finding was the statistically significant decrease of LNCaP cell viability in cells treated with TNF-*α* (100 ng/mL) (*P* < 0.05) for 24 h in comparison to control cells (Figure 1(a)) and in cells treated with TNF-*α* (10 and 100 ng/mL) (*P* < 0.001 and *P* < 0.001, resp.), IL-13 (20 and 100 ng/mL) (*P* = 0.002 and *P* = 0.003, resp.), or IL-1*β* (2 and 5 ng/mL) (*P* = 0.001 and *P* < 0.001, resp.) for 72 h in comparison to control cells (ctrl) (Figure 1).

No statistically significant decrease of PC-3 cell viability was observed in cells treated with IL-13, IFN-*γ*, IL-1*β*, or TNF-*α* in comparison to control cells (in 24 and 72 h) (data not shown).

### 3.2. Flow Cytometry Using Propidium Iodide Staining

Flow cytometry experiments were performed to analyze the effects of IL-13, IFN-*γ*, or IL-1*β* on cell cycle and death of LNCaP and PC-3 cells. The TNF-*α* was used as control because of its known procell death effects on LNCaP but not on PC-3 cells [11, 12]. The analysis of MAPK, PI3-K/Akt, and NF-*κ*B signaling pathways was performed on the basis of our previous study [35]. Briefly, the IL-13, IFN-*γ*, or IL-1*β*-induced effects on cell cycle and death were comparatively analyzed in LNCaP and PC-3 cells with and without pretreatment with the inhibitors of JNK (SP600125), p38 (SB203580), ERK 1/2 (UO126), PI3-K (LY-294002), and NF-*κ*B (BAY-117082) pathways.

#### 3.2.1. Effects of IL-13, IFN-*γ*, IL-1*β*, and TNF-*α* on LNCaP and PC-3 Cell Death

**Figure 2:**
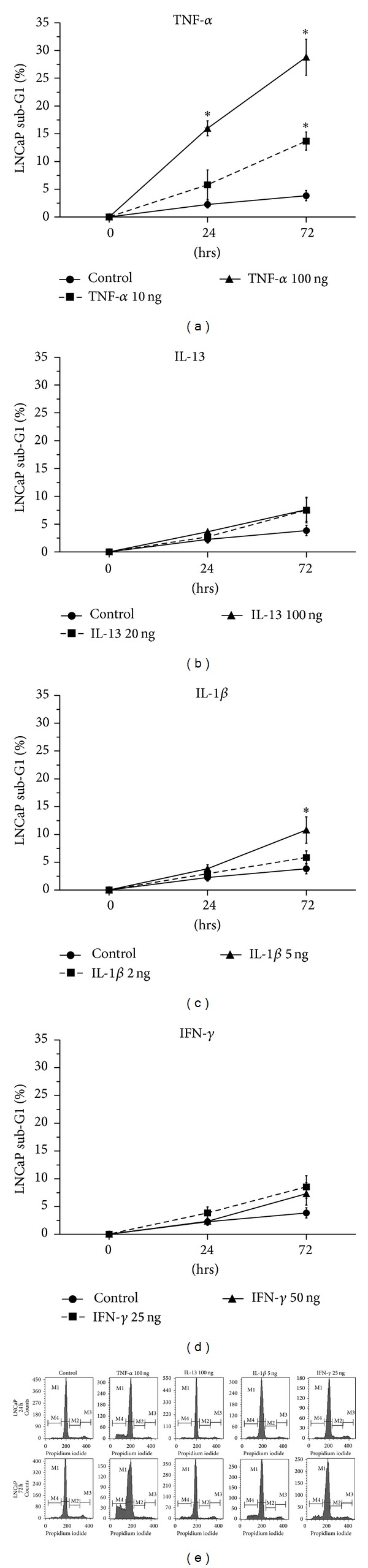
Flow cytometry analysis of LNCaP cells using PI staining. Time- and dose-dependent effects of (a) TNF-*α* (10 and 100 ng/mL), (b) IL-13 (20 and 100 ng/mL), (c) IL-1*β* (2 and 5 ng/mL), (d) IFN-*γ* (25 and 50 ng/mL) on LNCaP cell death (sub-G1 fraction) after treatment for 24 h or 72 h (*P < 0.05), and (e) representative histograms of cell distribution according to their DNA content, as determined by flow cytometry using PI staining (M1 represents G1 phase, M2 S phase, M3 G2/S phase, and M4 sub-G1 fraction).

Statistically significant increase of LNCaP cell death was observed in cells treated with TNF-*α* (100 ng/mL) for 24 h (*P* = 0.001) but not with TNF-*α* (10 ng/mL), IL-13 (20 and 100 ng/mL), IFN-*γ* (25 and 50 ng/mL), or IL-1*β* (2 ng/mL) for 24 h in comparison to control cells (Figure 2). The increase of LNCaP cell death was in the limits of statistical significance in cells treated with IL-1*β* (5 ng/mL) for 24 h (*P* = 0.053) (Figure 2).

A notable finding was the statistically significant increase of LNCaP cell death in cells treated with IL-1*β* (5 ng/mL) (*P* = 0.025) or TNF-*α* (10 and 100 ng/mL) for 72 h (*P* = 0.019 and *P* = 0.003, resp.) but not in cells treated with IL-13 (20 and 100 ng/mL), IFN-*γ* (25 and 50 ng/mL), or IL-1*β* (2 ng/mL) for 72 h in comparison to control cells (Figure 2).

**Figure 3:**
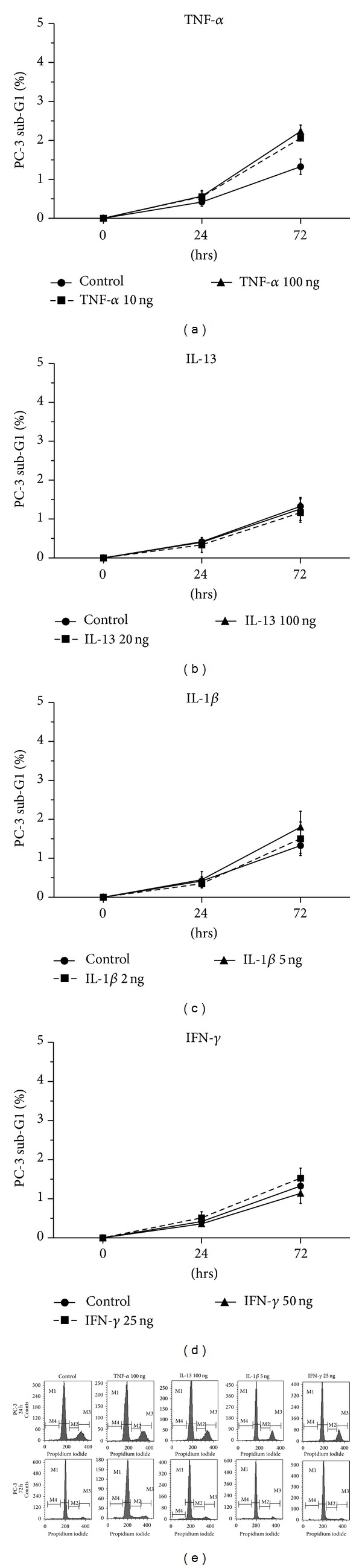
Flow cytometry analysis of PC-3 cells using PI staining. Time- and dose-dependent effects of (a) TNF-*α* (10 and 100 ng/mL), (b) IL-13 (20 and 100 ng/mL), (c) IL-1*β* (2 and 5 ng/mL), (d) IFN-*γ* (25 and 50 ng/mL) on PC-3 cell death (sub-G1 fraction) after treatment for 24 h or 72 h, and (e) representative histograms of cell distribution according to their DNA content, as determined by flow cytometry using PI staining. (M1 represents G1 phase, M2 S phase, M3 G2/S phase, and M4 sub-G1 fraction).

No statistically significant alterations of PC-3 cell death were observed in cells treated with (a) IL-13, IL-1*β*, or IFN-*γ*, (b) the inhibitors UO126 (inhibitor of ERK 1/2), SB203580 (inhibitor of p38), SP600125 (inhibitor of JNK), LY-294002 (inhibitor of PI3-K), and BAY 11-7082 (inhibitor of NF-*κ*B), and (c) the combinations of the cytokines IL-13, IL-1*β*, or IFN-*γ* with the inhibitors UO126, SB203580, SP600125, LY-294002, or BAY 11-7082 in comparison to the control (Figure 3).

#### 3.2.2. Effects of Inhibitors of NF-*κ*B, PI3-K, JNK, p38, and ERK 1/2 Pathways on LNCaP and PC-3 Cell Death

**Figure 4:**
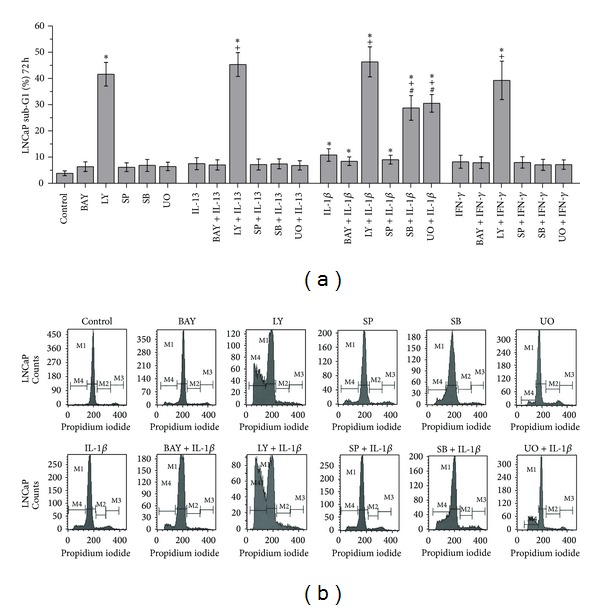
Flow cytometry analysis of LNCaP cells using PI staining. (a) Effects of IL-13 (20 ng/mL), IL-1*β* (5 ng/mL), IFN-*γ* (25 ng/mL), LY-294002 (20 *μ*M), BAY 11-7082 (30 *μ*M) SP600125 (30 *μ*M), S*Β*203580 (30 *μ*M), and UO126 (30 *μ*M) on LNCaP cell death (sub-G1 fraction). Cells were left untreated for 72 h (ctrl), or treated with the indicated cytokine or inhibitor alone for 72 h, or pretreated with the indicated inhibitor for 1 h and subsequently treated with the indicated cytokine for a total of 72 h. Statistically significant differences are depicted as follows: (∗) cytokine* versus* ctrl or inhibitor* versus* ctrl or inhibitor and cytokine* versus* ctrl (*P* < 0.05), (+) inhibitor and cytokine* versus* cytokine (*P* < 0.05), (#) inhibitor and cytokine* versus* inhibitor (*P* < 0.05). (b) Representative histograms of cell distribution according to their DNA content, as determined by flow cytometry using PI staining (M1 represents G1 phase, M2 S phase, M3 G2/S phase, and M4 sub-G1 fraction).

Treatment of LNCaP cells with LY-294002 (inhibitor of PI3-K) resulted in statistically significant increase of cell death in comparison to control cells (*P* = 0.001) (Figure 4). On the other hand, treatment of LNCaP cells with BAY 11-7082 (inhibitor of NF-*κ*B), SP600125 (inhibitor of JNK), SB203580 (inhibitor of p38), or UO126 (inhibitor of ERK 1/2) did not result in statistically significant alterations of cell death in comparison to control cells (Figure 4).

Noteworthy was the finding that pretreatment of LNCaP cells with SB203580 (inhibitor of p38) or UO126 (inhibitor of ERK 1/2) prior to treatment with IL-1*β* resulted in statistically significant increase of cell death in comparison (a) to cells treated with IL-1*β* alone (*P* = 0.010 and *P* = 0.002, resp.) and (b) to cells treated with SB203580 (inhibitor of p38) or UO126 (inhibitor of ERK 1/2) alone (*P* = 0.006 and *P* = 0.002, resp.) (Figure 4). On the other hand, pretreatment of LNCaP cells with SB203580 (inhibitor of p38) or UO126 (inhibitor of ERK 1/2) prior to treatment with IL-13 or IFN-*γ* did not result in statistically significant increase of cell death in comparison (a) to cells treated with IL-13 or IFN-*γ* alone and (b) to cells treated with SB203580 or UO126 alone (Figure 4).

Notable was also the finding that pretreatment of LNCaP cells with LY-294002 (inhibitor of PI3-K) prior to treatment with IL-13, IFN-*γ*, or IL-1*β* (a) resulted in statistically significant increase of cell death in comparison to cells treated with IL-13, IFN-*γ*, or IL-1*β* alone (*P* = 0.001, *P* = 0.011, and *P* = 0.003, resp.) and (b) did not result in statistically significant increase of cell death in comparison to cells treated with LY-294002 alone (Figure 4). On the other hand, pretreatment of LNCaP cells with BAY 11-7082 (inhibitor of NF-*κ*B) or SP600125 (inhibitor of JNK) prior to treatment with IL-13, IFN-*γ*, or IL-1*β* did not result in statistically significant alterations of cell death in comparison (a) to cells treated with IL-13, IFN-*γ*, or IL-1*β* alone and (b) to cells treated with BAY 11-7082 (inhibitor of NF-*κ*B) or SP600125 (inhibitor of JNK) alone (Figure 4).

**Figure 5:**
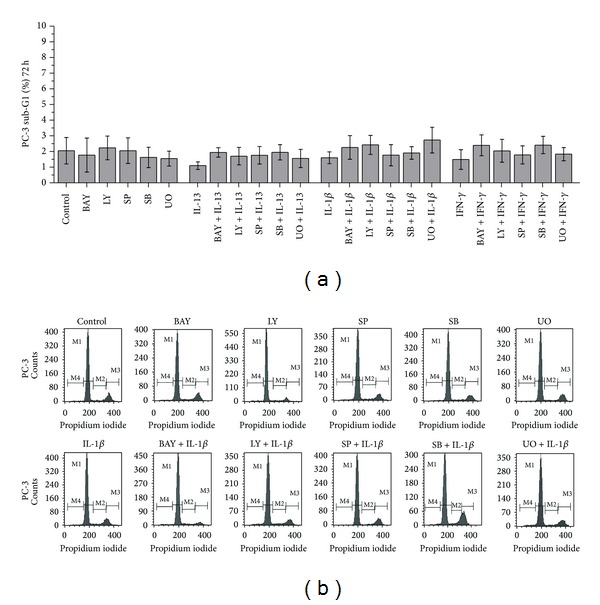
Flow cytometry analysis of PC-3 cells using PI staining. (a) Effects of IL-13 (20 ng/mL), IL-1*β* (5 ng/mL), IFN-*γ* (25 ng/mL), LY-294002 (20 *μ*M), BAY 11-7082 (30 *μ*M) SP600125 (30 *μ*M), S*Β*203580 (30 *μ*M), and UO126 (30 *μ*M) on PC-3 cell death (sub-G1 fraction). Cells were left untreated for 72 h (ctrl), or treated with the indicated cytokine or inhibitor alone for 72 h, or pretreated with the indicated inhibitor for 1 h and subsequently treated with the indicated cytokine for a total of 72 h. (b) Representative histograms of cell distribution according to their DNA content, as determined by flow cytometry using PI staining (M1 represents G1 phase, M2 S phase, M3 G2/S phase, and M4 sub-G1 fraction).

Treatment of PC-3 cells with LY-294002 (inhibitor of PI3-K), BAY 11-7082 (inhibitor of NF-*κ*B), SP600125 (inhibitor of JNK), SB203580 (inhibitor of p38), or UO126 (inhibitor of ERK 1/2) did not induce statistically significant alterations of cell death in comparison to control cells (Figure 5).

Pretreatment of PC-3 cells with LY-294002 (inhibitor of PI3-K), BAY 11-7082 (inhibitor of NF-*κ*B), SP600125 (inhibitor of JNK), SB203580 (inhibitor of p38), or UO126 (inhibitor of ERK 1/2) prior to treatment with IL-13, IFN-*γ*, or IL-1*β* did not induce statistically significant alterations of cell death in comparison to cells treated with (a) IL-13, IFN-*γ*, or IL-1*β* alone and (b) LY-294002 (inhibitor of PI3-K), BAY 11-7082 (inhibitor of NF-*κ*B), SP600125 (inhibitor of JNK), SB203580 (inhibitor of p38), or UO126 (inhibitor of ERK 1/2) alone (Figure 5).

### 3.3. Flow Cytometry Using Annexin V/PI Staining

**Figure 6:**
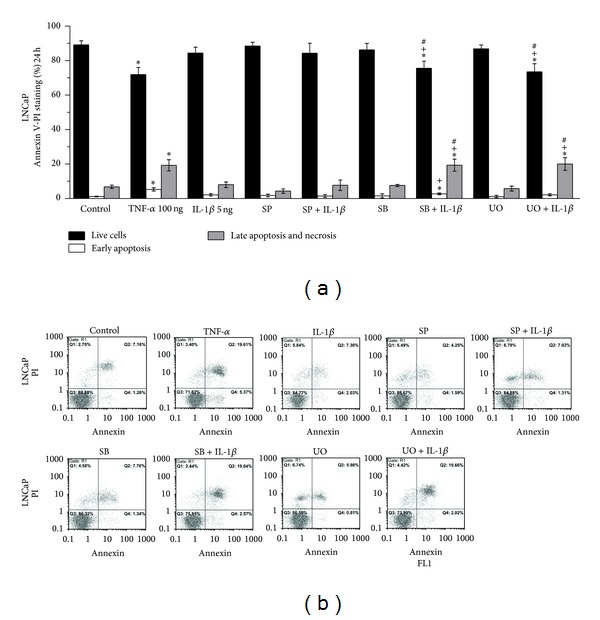
Flow cytometry analysis of LNCaP cells using annexin V/PI staining. (a) Effects of TNF-*α* (100 ng/mL), IL-1*β* (5 ng/mL), SP600125 (30 *μ*M), S*Β*203580 (30 *μ*M), and UO126 (30 *μ*M) on apoptosis and necrosis of LNCaP cells. Cells were left untreated for 24 h (ctrl), or treated with the indicated cytokine or inhibitor alone for 24 h, or pretreated with the indicated inhibitor for 1 h and subsequently treated with the indicated cytokine for a total of 24 h (statistically significant differences are depicted as follows: (∗) cytokine* versus* ctrl or inhibitor* versus* ctrl or inhibitor and cytokine* versus* ctrl (*P* < 0.05), (+) inhibitor and cytokine* versus* cytokine (*P* < 0.05), (#) inhibitor and cytokine* versus* inhibitor (*P* < 0.05)). (b) Representative histograms of cell distribution according to PI and annexin V staining as determined by flow cytometry (Q1 represents necrotic cells, Q2 late apoptotic and necrotic cells, Q3 live cells, and Q4 early apoptotic cells).

**Figure 7:**
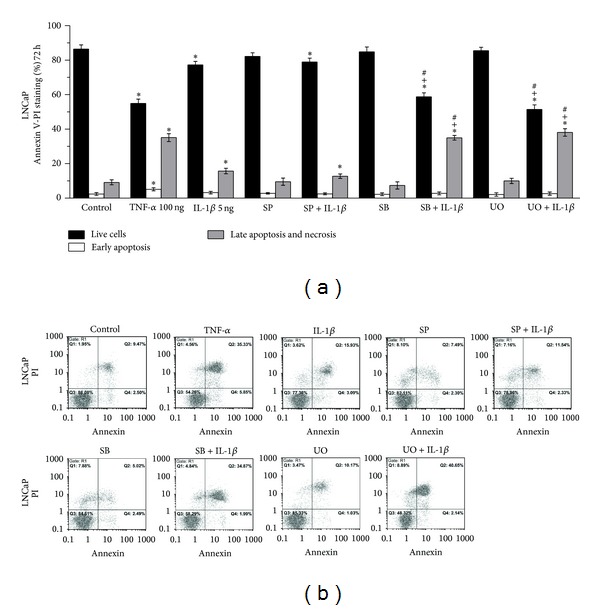
Flow cytometry analysis of LNCaP cells using annexin V/PI staining. (a) Effects of TNF-*α* (100 ng/mL), IL-1*β* (5 ng/mL), SP600125 (30 *μ*M), S*Β*203580 (30 *μ*M), and UO126 (30 *μ*M) on apoptosis and necrosis of LNCaP cells. Cells were left untreated for 72 h (ctrl), or treated with the indicated cytokine or inhibitor alone for 72 h, or pretreated with the indicated inhibitor for 1 h and subsequently treated with the indicated cytokine for a total of 72 h (Statistically significant differences are depicted as follows: (∗) cytokine* versus* ctrl or inhibitor* versus* ctrl or inhibitor and cytokine* versus* ctrl (*P* < 0.05), (+) inhibitor and cytokine* versus* cytokine (*P* < 0.05), (#) inhibitor and cytokine* versus* inhibitor (*P* < 0.05)). (b) Representative histograms of cell distribution according to PI and annexin V staining as determined by flow cytometry (Q1 represents necrotic cells, Q2 late apoptotic and necrotic cells, Q3 live cells, and Q4 early apoptotic cells).

Statistically significant increase of early apoptosis (Q4 fraction in the histogram) and late apoptosis and necrosis (Q2 fraction in the histogram) was observed in LNCaP cells treated with TNF-*α* in comparison to control cells (ctrl) for 24 h (*P* = 0.015 and *P* = 0.016, resp.) (Figure 6) and 72 h (*P* < 0.001 and *P* < 0.001, resp.) (Figure 7). Statistically significant increase of late apoptosis and necrosis levels (Q2 fraction in the histogram) was observed in LNCaP cells treated with IL-1*β* for 72 h in comparison to control cells (*P* = 0.006) (Figure 7).

Pretreatment of LNCaP cells with S*Β*203580 (inhibitor of p38) or UO126 (inhibitor of ERK 1/2) for 1 h prior to treatment with IL-1*β* for a total of 24 or 72 h resulted in statistically significant increase of late apoptosis and necrosis (Q2 fraction in the histogram) in comparison (a) to control cells ((*P* = 0.017 and *P* = 0.019, resp., for 24 h) (Figure 6) and (*P* < 0.001 and *P* < 0.001, resp., for 72 h) (Figure 7)), (b) to cells treated with IL-1*β* alone ((*P* = 0.019 and *P* = 0.017, resp., for 24 h) (Figure 6) and (*P* < 0.001 and *P* < 0.001, resp., for 72 h) (Figure 7)), and (c) to cells treated with SB203580 or UO126 alone ((*P* = 0.025 and *P* = 0.013, resp., for 24 h) (Figure 6) and (*P* < 0.001 and *P* < 0.001, respectively, for 72 h) (Figure 7)).

Pretreatment of LNCaP cells with SP600125 (inhibitor of JNK) for 1 h prior to treatment with IL-1*β* for a total of 72 h resulted in significant increase of late apoptosis and necrosis (Q2 fraction in the histogram) in comparison to control cells (*P* = 0.035). This cell death increase, however, was not statistically significant in comparison to that observed in cells treated with IL-1*β* or SP600125 alone (Figure 7).

### 3.4. Western Blot

**Figure 8:**
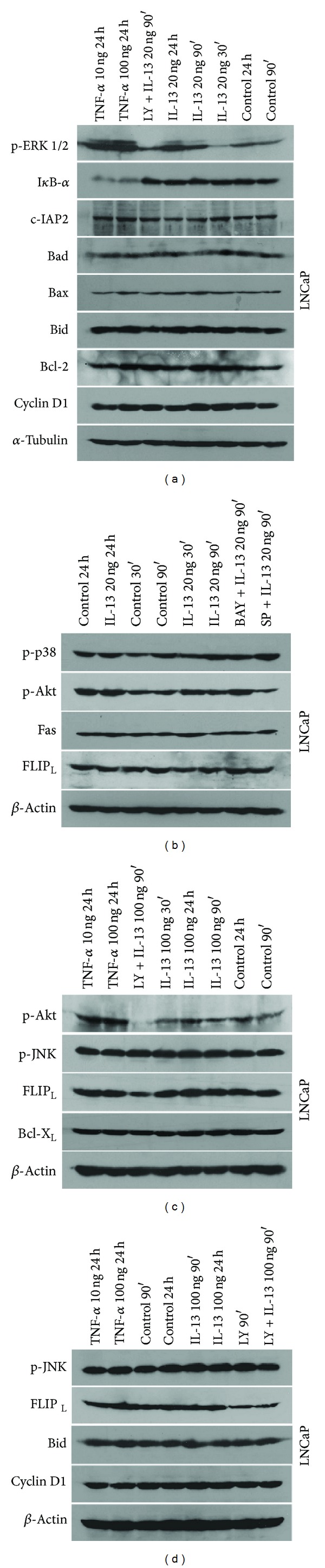
Western blot analysis of LNCaP protein expression. (a), (b), (c), and (d) LNCaP cells were treated with TNF-*α* (10 and 100 ng/mL) for 24 h or IL-13 (20 and 100 ng/mL) for 30 and 90 min and 24 h or LY-29004 (20 *μ*M) for 90 min or pretreated with LY-29004 (20 *μ*M), BAY-117082 (30 *μ*M), or SP600125 (30 *μ*M) for 1 h and then treated with IL-13 (20 and 100 ng/mL) for 90 min. Untreated cells for 30 and 90 min and 24 h were used as control (ctrl). Cell extracts were resolved by SDS-PAGE and analyzed by western blot with antibodies against Bid, Bad, Bax, Bcl-2, Cyclin D1, p-ERK 1/2, I*κ*B-*α*, c-IAP2, p-p38, p-Akt, Fas, FLIP_L_, p-JNK, and Bcl-X_L_. Reprobing with antibody against *α*-tubulin or *β*-actin was used as a loading and transfer marker.

**Figure 9:**
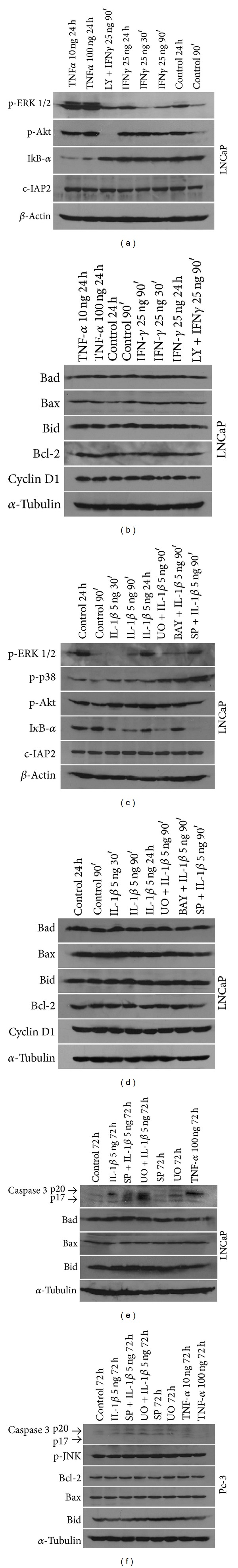
Western blot analysis of LNCaP protein expression. (a) and (b) LNCaP cells were treated with TNF-*α* (10 and 100 ng/mL) for 24 h or IFN-*γ* (25 ng/mL) for 30, 90 min, and 24 h or pretreated with LY-29004 (20 *μ*M) for 1 h and then treated with IFN-*γ* (25 ng/mL) for 90 min. Untreated cells for 90 min and 24 h were used as controls (ctrl). (c) and (d) LNCaP cells were treated with IL-1*β* (5 ng/mL) for 30, 90 min, and 24 h or pretreated with UO126 (30 *μ*M), BAY-117082 (30 *μ*M), or SP600125 (30 *μ*M) for 1 h and then treated with IL-1*β* (5 ng/mL) for 90 min. Untreated cells for 90 min and 24 h were used as controls (ctrl). (e) and (f) LNCaP and PC-3 cells were treated with IL-1*β* (5 ng/mL) or TNF-*α* (10 and 100 ng/mL) or SP600125 (30 *μ*M) or UO126 (30 *μ*M) for 72 h, or pretreated with SP600125 (30 *μ*M) or UO126 (30 *μ*M) for 1 h and then treated with IL-1*β* (5 ng/mL) for 72 h. Untreated cells for 72 h were used as controls (ctrl). Cell extracts were resolved by SDS-PAGE and analyzed by western blot with antibodies against Bid, Bad, Bax, Bcl-2, Cyclin D1, p-ERK 1/2, p-p38, p-Akt, I*κ*B-*α*, c-IAP2, and caspase 3. Reprobing with antibody against *α*-tubulin or *β*-actin was used as a loading and transfer marker.

We analyzed the expression patterns of p-ERK 1/2, p-p38, p-JNK, p-Akt, and *Ικ*B*α* in control cells and in cells treated with IL-13, IFN-*γ*, IL-1*β*, or TNF-*α*. Notable findings were (a) the time-dependent increase of p-ERK 1/2 and p-p38 in control, IL-13, IFN-*γ*, or IL-1*β* treated LNCaP cells (Figures 8 and 9) and (b) the time-dependent expression of p-Akt on control, IL-13, IL-1*β*, or IFN-*γ* treated LNCaP cells (Figures 8 and 9). On the other hand, expression of p-JNK was observed in control LNCaP cells and was unaffected by IL-13, IFN-*γ*, IL-1*β*, or TNF-*α* treatment (Figure 8).

Another notable finding was the detection of decreased *Ικ*B*α* expression in LNCaP cells treated with IL-1*β* or TNF-*α* whereas the levels of *Ικ*B*α* expression in IL-13 or IFN-*γ* treated cells were similar to those of control untreated cells (Figures 8 and 9). Reduced *Ικ*B*α* expression indicates NF-*κ*B activation [11, 12, 27, 32]. Increased levels of active caspase 3 were detected in LNCaP cells treated with TNF-*α* or pretreated with SP600125 (JNK inhibitor) or UO126 (ERK 1/2 inhibitor) prior to IL-1*β* treatment in comparison to control cells (Figure 9).

We also analyzed the expression patterns of major cell cycle and apoptosis regulators such as Cyclin D1, Bcl-2, Bcl-X_L_, Bax, Bad, Bid, Fas, c-FLIP_L_, c-IAP2, and c-IAP1. No notable alterations of the expression levels of Cyclin D1 (Figures 8 and 9), Bcl-2 (Figures 8 and 9), Bcl-X_L_ (Figure 8), Bax (Figures 8 and 9), Bad (Figures 8 and 9), Bid (Figures 8 and 9), Fas (Figure 8), c-FLIP_L_ (Figure 8), c-IAP2 (Figures 8 and 9), and c-IAP1 were detected between control and LNCaP cells treated with IL-13, IFN-*γ*, IL-1*β*, or TNF-*α*.

Noteworthy were the findings that, in comparison to LNCaP cells treated with IL-13 alone, (a) pretreatment with LY-294002 (PI3-K inhibitor) prior to IL-13 treatment resulted in reduction of p-Akt and c-FLIP_L_ and (b) pretreatment with SP600125 (JNK inhibitor) prior to IL-13 treatment resulted in reduction of p-Akt (Figure 8). On the other hand, in comparison to LNCaP cells treated with IL-13 alone, (a) pretreatment with LY-294002 (PI3-K inhibitor) prior to IL-13 treatment resulted in no changes in p-ERK 1/2, p-JNK, and *Ικ*B*α* expression (Figure 8), (b) pretreatment with BAY-117082 (NF-*κ*B inhibitor) prior to IL-13 treatment resulted in no changes in p-p38, p-JNK, p-Akt, and *Ικ*B*α* expression (Figure 8) and (c) pretreatment with SP600125 (JNK inhibitor) prior to IL-13 treatment resulted in no changes in p-p38, p-JNK, and *Ικ*B*α* expression (Figure 8).

Noteworthy were also the findings that, in comparison to LNCaP cells treated with IL-1*β* alone, (a) pretreatment with UO126 (ERK 1/2 inhibitor) prior to IL-1*β* treatment resulted in increase of p-p38 expression and decrease of *Ικ*B*α* expression (Figure 9), (b) pretreatment with BAY-117082 (NF-*κ*B inhibitor) prior to IL-1*β* treatment resulted in increase of p-p38 expression (Figure 9), and (c) pretreatment with SP600125 (JNK inhibitor) prior to IL-1*β* treatment resulted in increase of p-p38 expression and decrease of *Ικ*B*α* and p-Akt expression (Figure 9).On the other hand, in comparison to LNCaP cells treated with IL-1*β* alone, (a) pretreatment with UO126 (ERK 1/2 inhibitor) prior to IL-1*β* treatment resulted in no changes of p-ERK 1/2, p-Akt, and p-JNK expression (Figure 9), (b) pretreatment with BAY-117082 (NF-*κ*B inhibitor) prior to IL-1*β* treatment resulted in no changes of p-ERK 1/2, p-JNK, p-Akt, and *Ικ*B*α* expression (Figure 9), and (c) pretreatment with SP600125 (JNK inhibitor) prior to IL-1*β* treatment resulted in no changes of p-ERK 1/2 and p-JNK expression (Figure 9).

Pretreatment of LNCaP cells with LY-294002 (PI3-K inhibitor) prior to IFN-*γ* treatment resulted in no changes in p-ERK 1/2, p-JNK, and *Ικ*B*α* expression and reduction of p-Akt expression in comparison to cells treated with IFN-*γ* alone (Figure 9).

In PC-3 cells, no notable alterations on the expression levels of p-JNK, Bcl-2, Bax, Bid, or active caspase 3 were detected between control and cells treated with IL-1*β*, TNF-*α*, UO126, or SP600125 or cells pretreated with UO126 or SP600125 prior to IL-1*β* treatment (Figure 9).

## 4. Discussion

Cytokines play important roles in the regulation of the immunity, the inflammatory response, and the cell growth and death of normal and malignant cells [20, 38–42]. The effects of the cytokines TNF-*α*, TRAIL, IFN-*γ*, IFN-*α*, IFN-*β*, IL-13, and IL-1*β* on cell death and/or cell viability have been investigated in various prostate carcinoma cell lines such as LNCaP, PC-3, DU-145, and M12 [8–17, 43]. However, to the best of our knowledge, (a) the effects of IL-13, IFN-*γ*, and IL-1*β* on cell viability, cycle, and death of LNCaP and PC-3 cells and (b) the involvement of MAPK, PI3-K/Akt, and NF-*κ*B signaling pathways in these cytokine-induced cellular effects have not been systematically analyzed.

The major finding of the present study is that the inhibition of ERK 1/2 and p38 signaling pathways by the inhibitors UO126 and SB203580, respectively, caused a significant increase of the LNCaP cell death induced by IL-1*β* in comparison to LNCaP cells treated with IL-1*β*, UO126, or SB203580 alone. This cell death was found to be both apoptotic and necrotic using flow cytometry with annexin V/PI staining and western blot analysis that showed caspase 3 activation. Our data can be paralleled with previous findings showing that inhibition of p38 signaling (pretreatment with 10 *μ*M of the inhibitor SB203580 for 6 h) caused a significant increase of the LNCaP cell death induced by TNF-*α* (50 and 75 ng/mL for 48 h) in comparison to LNCaP cells treated with TNF-*α* or SB203580 alone for 48 h [10]. Collectively, the aforementioned results indicate that ERK 1/2 and p38 pathways may have protective roles against IL-1*β* and TNF-*α* induced LNCaP cell death. Therefore, together with a previous study [10], it could be suggested that the potentiation of LNCaP cell death by ERK 1/2 and p38 inhibition points to these molecules as possible targets for the treatment of androgen-dependent prostate cancer. Furthermore, we observed (a) that the constitutive expression of p-JNK in control LNCaP cells exhibited no notable alterations after IL-13, IFN-*γ*, or IL-1*β* treatment and (b) that the expression of p-ERK 1/2 and p-p38 is time dependent showing no notable alterations between control and IL-13, IFN-*γ*, or IL-1*β* treated LNCaP cells. This latter can be paralleled with previous findings showing that treatment of M12 cells with IFN-*γ* (100 U/mL for 1 h) did not further increase p-ERK 1/2 expression in comparison to control cells [14]. However, treatment of DU-145 cells with IL-1*β* (10 ng/mL for 10 or 15 min) further activates p38, ERK 1/2, and JNK in comparison to control cells [33]. Collectively, the aforementioned findings indicate that IL-13, IFN-*γ*, and IL-1*β*-induced modulation of MAPK activation in prostate cancer is cell-type dependent.

Previous studies reported that Akt is constitutively activated in LNCaP cells acting as important survival factor in these cells [22, 24, 29]. In this respect, suppression of PI-3 K/Akt signaling markedly enhanced the apoptotic activity of TNF-*α* and TRAIL in LNCaP cells, as determined by flow cytometry [22]. Moreover, TNF-*α* and TRAIL did not induce notable alterations of p-Akt expression in LNCaP cells in comparison to control [22]. However, the effects of IL-13 and IFN-*γ* on p-Akt expression in LNCaP cells, to the best of our knowledge, have not been analyzed by western blot. We observed that the expression of p-Akt is time dependent showing no notable alterations between control and IL-13, IFN-*γ*, or IL-1*β* treated LNCaP cells. This can be paralleled with previous western blot findings showing that treatment with IL-1*β* (0.5, 1 and 5 ng/mL for 48 h) and IFN-*γ* (100 U/mL for 1 h) did not further increase p-Akt expression in comparison to control in LNCaP and M12 cells, respectively [14, 17]. Moreover, we found by flow cytometry that treatment of LNCaP cells with the PI-3 K inhibitor LY-294002 (20 *μ*M for 24 h) induces significant increase of LNCaP cell death in comparison to control cells. The specificity of the PI-3 K inhibition was confirmed in the present study by the notable reduction of p-Akt expression in LNCaP cells pretreated with LY-294002, as evidenced by western blot. Our findings are consistent with previous data showing that (a) treatment of LNCaP cells with LY-294002 (5 *μ*M for 24 h) induced notable increase of cell death (5.5% of cells in sub-G1 fraction versus 1.8% of control cells), as determined by flow cytometry [22] and (b) treatment of LNCaP cells with LY-294002 (60 *μ*M for 4–6 h) induced extensive DNA fragmentation (laddering) in LNCaP cells [24]. Collectively, the aforementioned findings (a) indicate that IL-13, IFN-*γ*, and IL-1*β* do not induce notable alteration of p-Akt expression in LNCaP cells and (b) further support the role of PI-3 K pathway as important survival factor in these cells.

Furthermore, we observed that treatment with the PI-3 K inhibitor LY-294002 reduced the expression of the antiapoptotic c-FLIP_L_ [44] in LNCaP but not in PC-3 cells. This is in keeping with previous western blot findings in LNCaP and DU145 cells [45, 46] and further supports the concept that PI-3 K/Akt signaling pathway regulates the expression of c-FLIP in prostate carcinomas. Moreover, the reduction of the expression of the antiapoptotic c-FLIP_L_ may also explain, at least in part, the potential therapeutic interest of inhibiting the PI-3 K/Akt signaling pathway in prostate cancer [30].

There is evidence that the NF-*κ*B signaling pathway is involved in cell cycle and apoptosis regulation of prostate cancer [21, 23, 27, 28, 47]. A previous study has shown that IL-1*β* activates NF-*κ*B in LNCaP cells on the basis of a significant increase of phosphorylated NF-*κ*B in LNCaP cells treated with IL-1*β* in comparison to control untreated cells [17]. Moreover, IL-1*β* activates NF-*κ*B in prostate tissue in mouse models [48, 49]. Since NF-*κ*B activation proceeds through ubiquitination and degradation of the I*κ*B-*α* protein [11, 12, 27, 32], reduced levels of I*κ*B-*α* protein indicate NF-*κ*B activation. We found that I*κ*B-*α* expression was reduced in IL-1*β* treated LNCaP cells being in control levels in cells treated with IL-13 or IFN-*γ* or BAY-117082 (NF-*κ*B inhibitor) prior to IL-1*β* treatment. These findings are in keeping with the data of Bouraoui et al. [17] who showed that IL-1*β* activates NF-*κ*B in LNCaP cells and suggested that NF-*κ*B activation may be involved in IL-1*β* induced LNCaP cell death. This can be paralleled with previous evidence that NF-*κ*B activation may have proapoptotic effects in TNF-*α*-treated LNCaP cells [27, 50] as well as in other cells [47, 51, 52].

In the present study, PC-3 cells treated with (a) IL-13, IL-1*β*, IFN-*γ*, or TNF-*α*, (b) the inhibitors UO126, SB203580, SP600125, LY-294002, or BAY 11-7082 and (c) the combinations of IL-13, IL-1*β*, or IFN-*γ* with these inhibitors did not show statistically significant alterations of cell death in comparison to the control. These findings indicate that IL-13, IL-1*β*, and IFN-*γ* do not have antitumor activity in the androgen-independent PC-3 cells. Moreover, our findings concur with previous (a) flow cytometry data showing that the inhibitors LY-294002, PD98059, SB203580, and SP600125 do not induce significant alterations of PC-3 cell death [10, 22], (b) DNA data showing that LY-294002 does not induce DNA fragmentation (laddering) in PC-3 cells [24], and (c) morphological and western blot data showing that inhibition of NF-*κ*B does not induce significant alterations of PC-3 cell death [21]. The aforementioned findings, taken together, suggest that inhibition of ERK 1/2, p38, JNK, PI3-K, or NF-*κ*B pathways alone and in combination with IL-13, IL-1*β*, or IFN-*γ* may not have a major impact in the induction of PC-3 cell death.

Collectively, our study and previous findings [10] raise the question why only IL-1*β* and TNF-*α* but not IL-13 or IFN-*γ* show significant increase of LNCaP cell death after inhibition of p38 or ERK 1/2 pathways. It was found that IL-1*β* and TNF-*α* but not IL-13 or IFN-*γ* induce significant increase of cell death and NF-*κΒ* activation in LNCaP cells (see present study, [8, 10, 17, 27, 32]). In addition, (a) activation of NF-*κΒ* may mediate TNF-*α* induced procell death effects in LNCaP cells [27], (b) activation of NF-*κΒ* by TNF-*α* + irradiation has proapoptotic effects in LNCaP cells [50], (c) activation of NF-*κΒ* is required for induction of apoptosis in DU-145 cells treated by the synthetic retinoid CD437 [47], and (d) induction of NF-*κΒ* by TNF-*α* and IL-1*β* has a proapoptotic role in pancreatic beta cells [52]. Moreover, inhibition of p38 or ERK 1/2 potentiated IL-1*β* or TNF-*α* induced LNCaP cell death (see present study, [10]). Furthermore, inhibition of ERK 1/2 is involved in increased apoptotic effects in LNCaP cells [6]. Therefore, on the basis of the aforementioned findings (see present study, [6, 8, 10, 17, 27, 32, 47, 50, 52]), we suggest that concomitant procell death effects due to (a) the IL-1*β* or TNF-*α* induced NF-*κ*B procell death activity in LNCaP cells and (b) the enhancement of IL-1*β* or TNF-*α* induced LNCaP cell death potential after inhibition of p38 or ERK 1/2 pathways may explain the findings that only IL-1*β* and TNF-*α* but not IL-13 or IFN-*γ* show significant increase of LNCaP cell death after inhibition of p38 or ERK 1/2 pathways (see present study, [10]).

## 5. Conclusions

The major finding of the present study was the significant increase of LNCaP cell death (apoptotic and necrotic) in cells treated with inhibitors of ERK 1/2 (UO126) and p38 (SB203580) prior to IL-1*β* treatment in comparison to cells treated with UO126, SB203580, or IL-1*β* alone. Significant increase of LNCaP but not PC-3 cell death was detected after treatment with LY-294002 (inhibitor of phosphatidylinositol 3-kinase) in comparison to control. No significant increase of LNCaP and PC-3 cell death was observed after treatment with SP600125 (inhibitor of JNK), SB203580 (inhibitor of p38), UO126 (inhibitor of ERK 1/2), or BAY 11-7082 (inhibitor of NF-*κ*B) in comparison to control. Reduced c-FLIP_L_ expression was observed in LNCaP cells treated with LY-294002. This further supports the concept that PI-3 K/Akt signaling pathway regulates c-FLIP expression in androgen-dependent prostate carcinomas. The significant potentiation of LNCaP cell death by inhibition of ERK 1/2, p38, and PI3-K pathways may provide a rationale for therapeutic approach in androgen-dependent prostate cancer.
